# Pandemic 2009 H1N1 virus infection in children and adults: A cohort study at a single hospital throughout the epidemic

**DOI:** 10.1186/1755-7682-5-13

**Published:** 2012-03-26

**Authors:** Jung-Woo Rhim, Eun-Ji Go, Kyung-Yil Lee, You-Sook Youn, Myung-Sook Kim, Sun Hee Park, Ji-Chang Kim, Jin-Han Kang

**Affiliations:** 1 Departments of Pediatrics, College of Medicine, The Catholic University of Korea, 505 Banpo-dong, Seocho-gu, Seoul 137-701, Republic of Korea; 2 Department of Pediatrics, The Catholic University of Korea, Daejeon St. Mary's Hospital, 520 Daeheung-dong, Jung-gu, Daejeon 301-723, Republic of Korea; 3 Department of Internal Medicine, The Catholic University of Korea, Daejeon St. Mary's Hospital, 520 Daeheung-dong, Jung-gu, Daejeon 301-723, Republic of Korea; 4 Department of Radiology, The Catholic University of Korea, Daejeon St. Mary's Hospital, 520 Daeheung-dong, Jung-gu, Daejeon 301-723, Republic of Korea

**Keywords:** H1N1 influenza virus, Epidemiology, Pneumonia, Children, Adults

## Abstract

**Background:**

In 2009, there was an influenza pandemic in South Korea. The aim of this study was to evaluate the epidemiological, clinical and laboratory characteristics of this infection in children and adults.

**Methods:**

We evaluated the epidemiologic characteristics of patients infected with the 2009 H1N1 influenza A virus (4,463 patients, age range from 2 mo to 86 y), and the clinical and laboratory findings of 373 inpatients (80/217 children, ≤ 15 y, had pneumonia and 36/156 adults, > 16 y, had pneumonia) in a single hospital during the epidemic.

**Results:**

The majority of infected patients (94%) were less than 40 y, and greater than 90% of cases occurred during a two-month period. The rates of admission and pneumonia were 8.4% (373/4,463) and 2.5% (116/4,463), respectively. The rates of admission and pneumonia, total duration of fever, the frequency of underlying diseases, and the values of C-reactive protein and erythrocyte sedimentation rate tended to increase as age increased; highest rates were found in the ≥ 65 y group. Pneumonia was founded more boys than girls in children, but more female than male in adults. The adult patients with pneumonia had higher leukocyte counts with lower lymphocyte differentials than the group without pneumonia, as shown in children group.

**Conclusion:**

Our results suggest that the immunologic reaction to viral insults may be associated with age, sex and underlying diseases, and that unknown herd immunity may affect populations. The patients with underlying diseases, especially in older patients may have immunologic insufficiency that is associated with immunologic consumption by the underlying diseases.

## Background

Since the 2009 H1N1 influenza A virus was first isolated in North America in spring 2009, there was an influenza pandemic in South Korea in 2009 as well as in other countries. Although the mortality of the 2009 H1N1 influenza A virus pandemic (2009 H1N1 flu) has been reported to be not exceed that of seasonal (inter-pandemic) influenza, some epidemiological characteristics of the pandemic, including its age distribution, differ from those of seasonal influenza [1,2]. In seasonal influenza, young infants and older persons are vulnerable and the mortality rate is higher in the extreme of age groups. However, in pandemic influenza occurring a 10-40 year cycle, people of all ages without immunity may be affected, and young healthy adults can be fatal due to pandemic influenza [1-4]. During the 20th century, 3 pandemics were documented: 1918 Spanish flu, 1957 Asian flu and 1968 Hong Kong flu. The 2009 H1N1 flu was the first pandemic in the 21st century [5]. In the 2009 H1N1 flu and in other pandemics, a majority of infected patients recovered from this infection without complications. However, some previously healthy patients developed pneumonia. Patients that developed severe pneumonia can experience acute respiratory distress syndrome (ARDS), multi-organ failure, and even death. Nevertheless, the mortality rate of the 2009 H1N1 flu was far less than that of previous pandemics [6-9].

Despite of many clinical and experimental studies, the pathogenesis of acute lung injury (pneumonia) in influenza infections remains unknown. Some experimental and clinical studies have suggested that the pathogenesis of acute lung injury in influenza infections is associated with excessive host response such as the cell-mediated immune reaction [10-12]. The immune system of the host matures through childhood and then declines as people age [13-15]. Thus, it could be postulated that the infants and the elderly have either an immature or reduced immune response to viral infection, respectively. Furthermore, this may explain the higher mortality rate in these age groups during the winter influenza season. However, we previously observed that pneumonia and severe pneumonia were more prevalent in the 5-9 y group than in the 0-4 y group [16].

Although the 2009 H1N1flu occurred nearly four decades after the 1968 Hong Kong flu, thanks to new diagnostic tools such as real-time reverse transcriptase-polymerase chain reaction (RT-PCR) and nationwide surveillance system, many investigators could more exactly evaluate the epidemiological and clinical characteristics regarding the 2009 H1N1 flu. In this study, we aimed to evaluate the epidemiological, clinical and laboratory features of patients infected with the 2009 H1N1 virus at a single hospital and to compare these parameters between age groups. Also, we tried to explain the reasons of the epidemiological characteristics of the 2009 H1N1 flu, including the lower mortality rate in healthy persons than in previous pandemics.

## Methods

Daejeon is one of the largest cities located central in South Korea, and its population is 1.48 millions. The Catholic University of Korea Daejeon St Mary's Hospital is one of 5 general hospitals (> 600 beds) in the city and has 670 beds for children and adults. During the epidemic in Korea as the same period of this study (September 1st, 2009 to January 31st, 2010), 5 general hospitals, including our institution in Daejeon, performed the primary care for the outpatient and inpatient cases according to the Korea government policy, as the strategic positional hospitals for 2009 H1N1 flu. Therefore, the policies of patient care, including use of diagnostic tool (RT-PCR) and antivirals (oseltmivir), were similar among general hospitals in large cities in Korea.

The subjects of this study were all patients who were positive diagnostic RT-PCR (AccuPower™ in Korea, BiONEER, Alameda, CA, USA) on nasopharyngeal and throat swabs, and visited our institution during epidemic period in Korea.

For the evaluation of epidemiological characteristics of 2009 H1N1 flu, a total of 4,463 patients were analysed. In addition, we reviewed the medical records and chest radiographic findings of 373 inpatients (217 children, ≤ 15 y and 156 adults, > 16 y) for the evaluation of clinical characteristics. To compare the clinical and laboratory findings according to age, we divided the subjects into 4 age groups: the 0-15 y group, the 16-40 y group, the 41-64 y group and the ≥ 65 y group. The patients that were less than or equal to 15 y were treated in the Department of Paediatrics, and the patients that were greater than 16 y were treated in the Department of Internal Medicine. Some data compiled in this study regarding the ≤ 15 group was previously published [16]. The first day of fever and/or severe respiratory symptoms such as dyspnea were regarded as the first day of illness. Fever was defined as greater than 38°C using an eardrum thermometer. Pneumonia was defined as any infiltration on chest radiographic findings with clinical symptoms such as fever and cough. The study was approved by the Institutional Review Board of the Catholic University of Korea, Daejeon St Mary's Hospital.

### Statistical analysis

The clinical and laboratory information of the admitted patients presented as the median and range (minimum to maximum). Statistical significance was assessed using the Student's *t*-test and the ANOVA test for continuous variables and the *χ*^2 ^test and the Linear by linear association method for categorical variables. The data were analysed using SPSS version 12.0 for Windows (SPSS Inc., Chicago, IL, USA), and a P value less than 0.05 was considered significant.

## Results

During the study period, a total of 9,269 patients with influenza-like illness were examined by RT-PCR, and 4,463 patients were positive. Among them, 373 patients, 217 children (≤ 15 y, 80 had pneumonia) and 156 adults (> 16 y, 36 had pneumonia, 100 patients in the 15-40 y, 35 patients in the 41-64 y and 21 patients in the > 65 y group), were hospitalized (Table 1).

**Table 1:** The admission rates and pneumonia cases according to age in the total cohort (n = 4,463)

Age groups	0-15 y (n = 2971)	16-40 y (n = 1222)	41-64y (n = 228)	≥ 65 y (n = 42)	*P*
% of total cases	66. 6	27.4	5.1	0.9	-

Male:female ratio	1.1:1	1:1.1	1:1.5	1:2	< 0.001

Admission rate (%, n)	7.3 (217)	8.2 (100)	15.4 (35)	50 (21)	< 0.001

Pneumonia (%, n)	2.7 (80)	1.3 (16)	3.9 (9)	26.2 (11)	0.003

### Epidemiological features of the patients with the 2009 H1N1 virus infection

**Figure 1:**
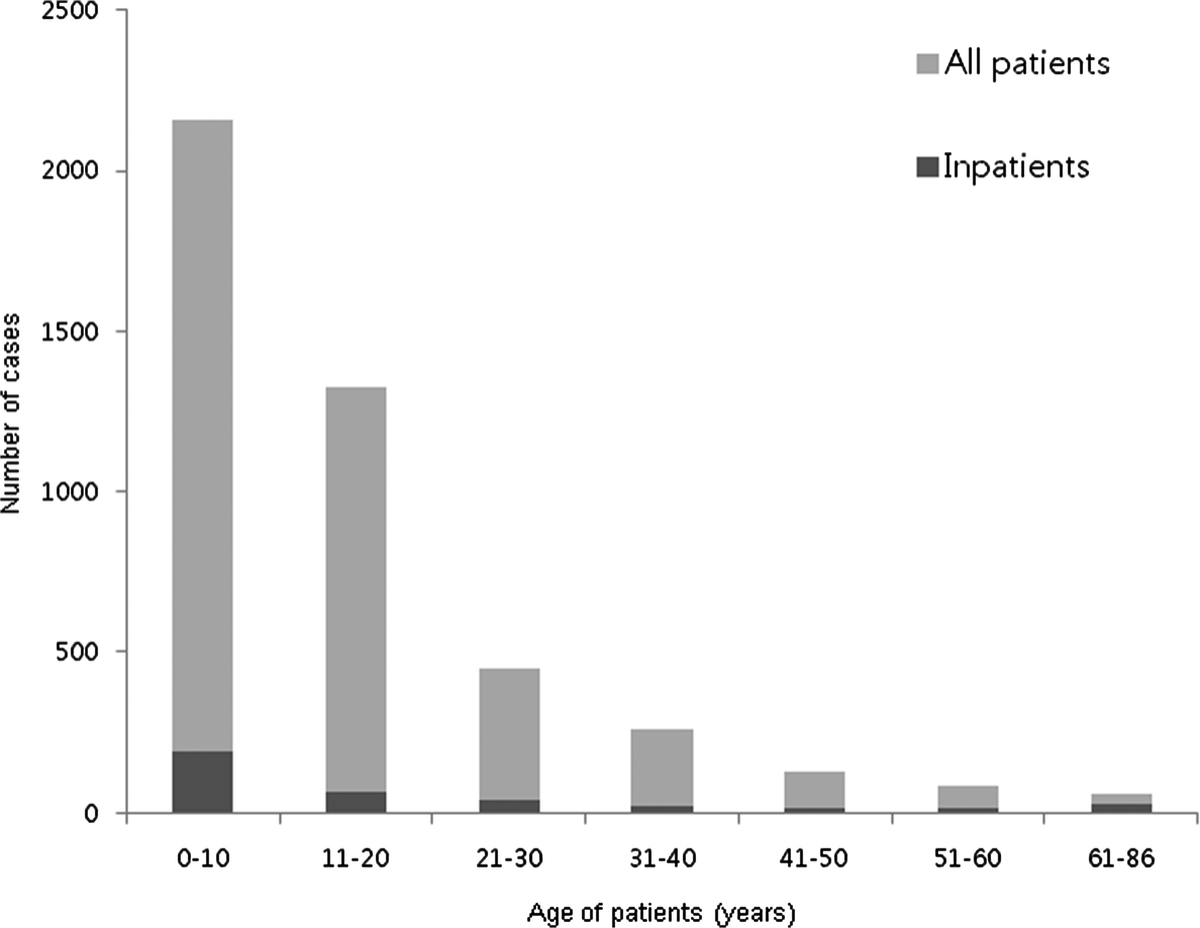
**Age distribution of the H1N1 virus infected patients in this study**.

**Figure 2:**
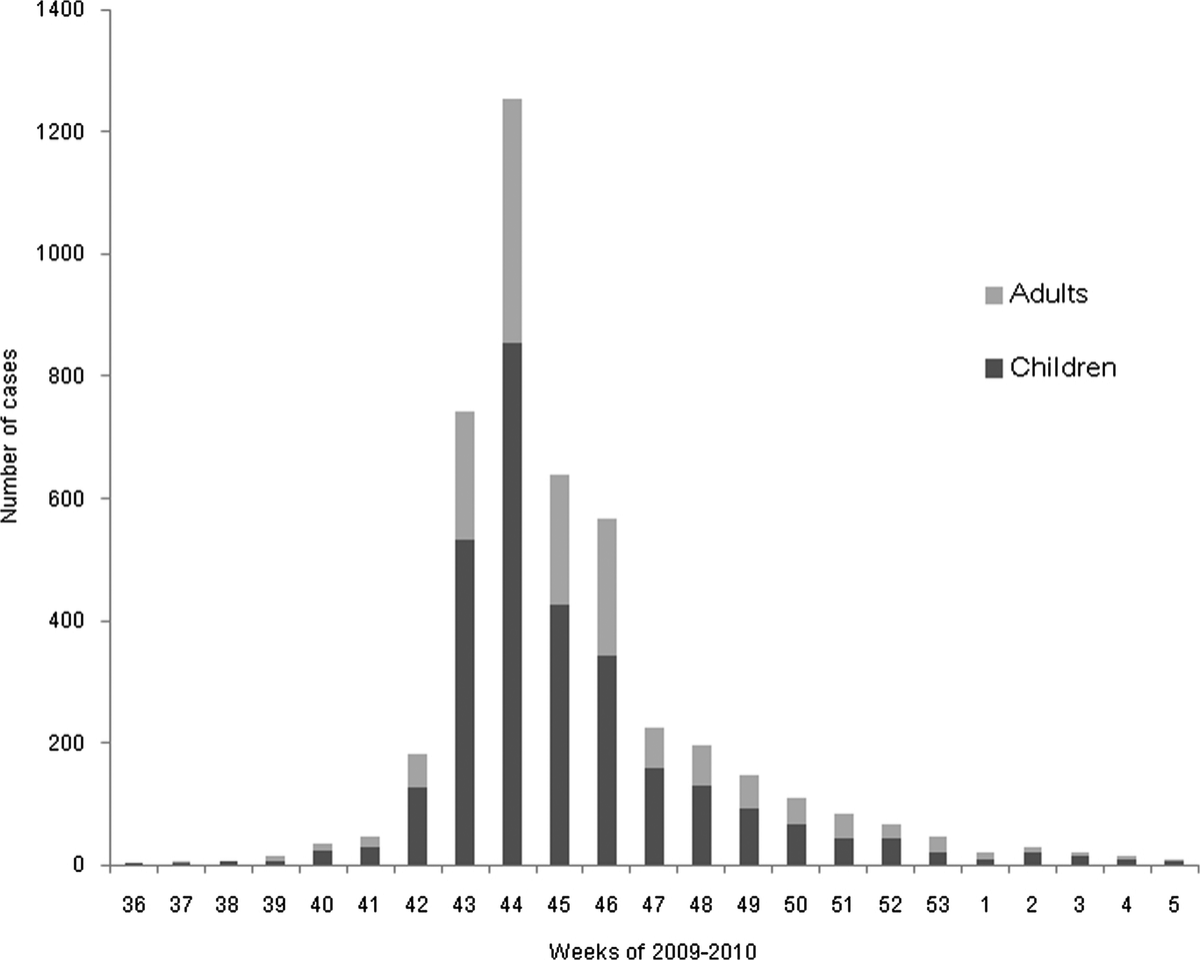
**Weekly frequency of H1N1 virus infection during study period**.

The median age of the patients (4,463 cases) was 11 y (range of 2 mo-86 y, mean 15 ± 13.5 y) and the male-to-female ratio was 1:1 (2,274/2,189). However, there were more boys in children group and more females in adult groups (Table 1). The age distribution of the patients is shown in Figure 1. In total, 48.4% of the patients were in the 0-10 y group (2,160), 29.8% were in the 11-20 y group (1,329), 10% were in the 21-30 y group (447), and 5.8% were in the 31-40 y group (258). Therefore, children and young adults comprised the majority of the patients (94%). There was a decreasing trend in the number of patients older than 41 y as increased age, and the ≥ 65 y group comprised only 0.9% (42) of the total patients (Figure 1 and Table 1). Over three quarters of the cases occurred during a single month (October 18th-November 14th) (Figure 2). This pattern was similar in both the children and adults (Figure 2, black and gray bars).

### Clinical features of the inpatients with the 2009 H1N1 virus infection

The median age of the 373 hospitalized patients was 10 y (range 2 mo- 86 y, mean 19.4 ± 20.1 y), and the male-to-female ratio was 1.1:1 (196/177). The total admission rate due to influenza infection was 8.4% (373/4,463). The age distribution of the patients admitted to hospital are shown on Figure 1 (black bar), and these demonstrated similar patterns to those of the total patient cohort except older age group. The admission rate of the 0-10 y was 8.8% (191/2,160), the 11-20 y was 4.7% (62/1,329), the 21-30 y was 9.2% (41/447), the 31-40 y was 8.9% (23/258), the 41-50 y was 12.1% (15/124), the 51-60 y was 16.7% (14/84), and the ≥ 61 y was 44.3% (27/61).

All inpatients received the recommended doses of oseltamivir and the majority of inpatients received a broad-spectrum antibiotic. In total, 338 patients (90.6%) received oseltamivir within 48 h of fever onset. Six adult patients (4 in the 41-65 y group and 2 in the ≥ 65 y group) with underlying diseases were infected during their hospital stay and these patients were excluded from the subjects. Additionally, four of the infected patients were pregnant women, and their clinical course was uneventful. We analysed the chest radiographs of the inpatients and found that 116 patients had pneumonia (80 children and 36 adults). Pneumonia was detected in 31.3% (116/373) of the admitted patients, and 2.6% (116/4,463) of the total infected patients, respectively (Table 1). No children were treated in the intensive care unit. However, six adult patients were treated in the intensive care unit, two had ARDS and 4 were at risk of deterioration because of underlying diseases. None of the infections in this study was fatal.

### Clinical and laboratory features according to age

We classified the admitted patients into 4 age groups, as mentioned previously. Interestingly, there were significant differences in the male-to-female ratios in the admitted patients and the pneumonia patients between children (≤ 15 y) and adults (16-86 y). In the admitted patients, the male-to-female ratio was 1.6:1(132:84) in children, whereas the ratio was 1:1.4 (64:92) in adults. Additionally, in the patients with pneumonia the male-to-female ratio was 3:1(60:20) for the children and 1:2(12:24) in the adults. There was a correlation between increased age and an increase in the admission rate and the frequency of underlying diseases (linear by linear association test), and the total duration of fever and the hospitalization (ANOVA test). In laboratory findings, the C-reactive protein (CRP) and erythrocyte sedimentation rate (ESR) values displayed similar correlation (ANOVA test) (Table 2). The ≥ 65 y group showed significant differences compared to other age groups in nearly all of the examined parameters (*χ*^2 ^test and Student's *t*-test, data not shown). The frequency of underlying diseases was significantly different among age groups. Many patients had more than one underlying diseases and these patients were more prevalent in older groups. The underlying diseases of the admitted patients are shown in Table 3.

**Table 2:** Clinical and laboratory findings of the admitted patients according to age (n = 373)

Age groups	0-15 y (n = 217)	16-40 y (n = 100)	41-64y (n = 35)	≥ 65 y (n = 21)	*P*
Clinical Characteristics					

Age, mean (y)	6.2 ± 3.7	25.2 ± 7	51.9 ± 6.7	74 ± 5	< 0.001

Male/female	132/85	45/55	14/21	5/16	< 0.001

Duration of fever (d)					

Before admission	2.0 ± 1.9	2.0 ± 1.1	1.7 ± 1.5	1.5 ± 1.3	0.045

Total duration	2.3 ± 1.0	2.9 ± 1.3	3.0 ± 1.7	4.1 ± 3.0	< 0.01

Oseltamivir, < 48 h*	205 (94)	89 (89)	29 (83)	15 (71)	NS

Hospitalisation (d)	4.9 ± 1.5	5.4 ± 2.2	5.2 ± 2.9	8.8 ± 5.1	< 0.001

Pneumonia					

Number (%)	80 (37)	16 (16)	9 (29)	11 (52)	NS

Male/female	60/20	4/11	4/5	3/8	< 0.001

Underlying diseases	17 (7.8)	15 (15)	15 (42.9)	16 (76.2)	< 0.001

Laboratory findings					

Haemoglobin (g/dL)	12.6 ± 0.9	13.5 ± 2.1	13.1 ± 1.6	12.9 ± 1.4	< 0.001

Leukocyte (×10^9^/L)	7.4 ± 3.6	6.4 ± 2.6	6.8 ± 2.8	7.6 ± 3.2	< 0.001

Neutrophil (%)	63 ± 19	66 ± 15	71 ± 14	64 ± 17	NS

Lymphocyte (%)	27 ± 17	23 ± 13	20 ± 12	24 ± 14	0.037

Monocyte (%)	9 ± 4	8 ± 4	8 ± 4	9 ± 4	NS

Platelet (×10^9^/L)	230 ± 59	202 ± 46	204 ± 51	198 ± 55	0.001

CRP (mg/dL)	1.6 ± 2.0	2.4 ± 3.8	3.8 ± 4.6	6.1 ± 6.9	< 0.001

ESR (mm/h)	17 ± 12	19 ± 17	37 ± 22	42 ± 19	< 0.001

CRP; C-reactive protein, ESR; erythrocyte sedimentation rate. NS, statistically non-specific

* Number (%) of patients who received oseltamivir treatment within 48 h of fever onset

**Table 3:** Underlying diseases of the admitted patients according to age

Age groups	0-15 y (n = 217)	16-40 y (n = 100)	41-64 y (n = 35)	≥ 65 y (n = 21)
Total cases (n,%)	17 (7.8)	15 (15)	15 (42.9)	16 (76.2)

Hypertension		1	2	6

Chronic lung disease				

COPD			3	7

Asthma	10	4	2	3

Others	1			2

Cancer		1	1	

Diabetes		3	2	3

Neurologic disorder	4	1	1	3

Heart disease		1	1	5

Chronic liver disease	1	2	4	3

Chronic renal disease	1	3	2	1

Autoimmune disorder		2	1	

Immunosuppresion	1	3	1	1

Pregnancy*		4		

COPD; chronic obstructive pulmonary disease

* Pregnancy was not defined as underlying disease

We previously found that children with pneumonia had higher leukocytes counts with lower lymphocyte differentials than the children without pneumonia [16]. The adult patients with pneumonia had higher leukocytes counts, CRP and ESR values with lower lymphocyte differentials than the adults without pneumonia (Table 4).

**Table 4:** Clinical and laboratory findings of adults patients (> 16 y) with and without pneumonia (n = 156)

Group	No Pneumonia (n = 120)	Pneumonia (n = 36)	*P*
Clinical characteristics			

Mean age (y)	35 ± 17	48 ± 22	0.002

Male/Female	53/67	11/25	NS

Duration of fever (d)			

Before admission	1.8 ± 1.2	2.1 ± 1.5	NS

Total	3.2 ± 2.9	3.9 ± 2.3	NS

Hospitalization (d)	5.2 ± 2.9	7.9 ± 3.8	< 0.001

Oseltamivir, < 48 h*	103 (86.7)	29 (80.6)	NS

Laboratory findings			

Haemoglobin (g/dL)	13.4 ± 2.0	12.5 ± 2.1	0.02

Leukocyte (×10^9^//L)	6.2 ± 2.4	7.9 ± 3.5	0.009

Neutrophil (%)	64 ± 15	76 ± 12	< 0.001

Lymphocyte (%)	24 ± 14	16 ± 9	< 0.001

Monocyte (%)	10 ± 4	7 ± 4	0.001

Platelet (×10^9^/L)	205 ± 50	201 ± 69	NS

CRP (mg/dL)	2.3 ± 4.0	6.6 ± 6.5	0.001

ESR (mm/h)	22 ± 19	35 ± 22	0.001

^a ^Number (%) of patients who received oseltamivir treatment within 48 h of fever onset

## Discussion

In this study, we evaluated the epidemiologic characteristic of the H1N1 virus infected patients of all ages in a single hospital throughout the epidemic. The age distribution pattern as shown in Figure 1 for our study has been observed in other regions of Korea [9]. Additionally, other countries found that children and young adults (< 40 y) comprised a predominant portion of the infected patient and that older adult groups (> 40-50 y) were possibly protected from this new viral infection [9,17-21]. Moreover, the mortality in this pandemic was not higher than for seasonal influenza and was highest in the older adult group, which is similar to seasonal influenza, although the numbers of infected patients in the older adult group was very small [7-9].

It is unknown what characteristics of the 2009 H1N1 flu caused the trends identified in this study. In Korea, because the rates of seasonal influenza vaccination during the recent decade was relatively even in the adult population with higher rates of vaccination in the under 5 y group and the older than 60 y group, it is unlikely that the vaccination status of the population was responsible for the age distribution of this pandemic. In addition, the results from serologic studies on the cross-reactive antibody response to the 2009 H1N1 virus before the pandemic in all age groups were different among the populations [22-25]. In Korea, prior to the pandemic the seroprevalence of antibodies that were cross-reactive to the H1N1 virus were 20.0% in the 19-59 y group and 27.3% in the older than 60 y group with no statistical differences among adult groups [23]. Dudareva et al. in Germany reported that those younger than 50 y group had highest levels of cross-reactive antibodies prior to the pandemic and the highest infection rates. Furthermore, they proposed that one of the possible reasons for the lower risk of infection among older persons could be pre-existing immunity not detectable by cross-reactive antibodies [24].

Given that this pandemic reemerged nearly 4 decades after last pandemic (1968 Hong Kong flu), it is possible that cross-immunity and/or an unknown herd immunity against a previous pandemic influenza virus (known as H3N2) may have played a role on populations in this pandemic, although the virus subtypes were different. Previous epidemiological studies on measles in the South Pacific islands, including Hawaii, have provided the new insights into new pathogens and herd immunity [26-28]. Epidemiologically, it is well documented that when a new pathogenic virus, such as the measles virus, is introduced into an immune-naive isolated population, nearly all inhabitants of all ages are affected, and that the severity of the disease and the mortality rate is very high (> 10-50%). In addition, the mortality is paradoxically the highest in the healthiest age-group (20-40 y) with the most active immune function. Then, in subsequent epidemics the severity of the disease begins to weaken because of unknown herd immunity [26,27]. In Faröe Islands, when the measles were re-introduced 65 years after the first disaster, the older than 65 population was protected from measles, and the mortality in this population was far less than the first attack [26,28]. Therefore, it is possible that the epidemiological characteristics of pandemic influenza are similar to those of measles. The severity of influenza in this pandemic was far less than that of the Spanish flu, which was a devastating disaster, because of the highest mortality rate in young adult groups. In addition, changing epidemiology has been observed for some viral infections, such as hemorrhagic fever with renal syndrome after the introduction of the vaccine in Korea [29], and acquired immunodeficiency syndrome (AIDS) after the introduction of antivirals [30]. An initially severe disease with high mortality can transform into a milder phenotype over time.

The characteristic explosive infection rate during the pandemic, which is shown in Figure 2, has also been reported in other regions of Korea and other countries [7-9,17-21]. This epidemiological pattern was observed in countries where vaccination for H1N1 virus was not performed, including Australia and New Zealand [17,18,21], and in countries where the vaccination started during the pandemic including Korea [9,19,20]. The rapid decline in the number of infection might also be explained by unknown herd immunity to pandemic influenza virus [16].

Although it was reported that a significant proportion of the populations can be infected with pandemic influenza, up to 40-50%, based on previous seroprevalence studies, there have been few population based data for infection rates during previous pandemics because of the lack of diagnostic tools such as RT-PCR [1,2,4]. In Korea during the 2009 H1N1 flu period, Korea Centers for Disease Control and Prevention reported that a total of 740,835 patients (1.5% of the 49 millions of total population in South Korea, 1,492 per100,000 persons) had been infected with H1N1 virus and confirmed by RT-PCR, and also reported that 225 of these patients died [9]. Therefore, there may have been many patients with asymptomatic or mild phenotypes in the 2009 H1N1 flu, regardless of the half RT-PCR positivity caused by febrile influenza-like illness. Follow-up seroprevalence studies conducted after the 2009 H1N1 flu have shown that greater than 40-50% of the examined samples from young adults were positive [24,25], and these findings support the possibility of the unknown herd immunity to pandemic influenza. In present cohort study, the calculated incidence of pneumonia in the 2009 pandemic was 2.6% of the infected patients with no fatal cases. The mortality rate in Korea was reported as 0.3 per 1,000 patients (0.03%), and other countries reported a range of mortality rates from 0.01 to 0.05% [7,8,17,19,21].

The male-to-female ratio was 1:1 for the total patients (4,463). However, in the inpatient group there was a higher number of boys than girls in the children (1.6:1) and a higher number of female than male in the adults (1:1.4). The male-to-female ratios among those with pneumonia showed a similar trend: 3:1 in the children and 1:2 in the adults. This tendency has been previously reported by studies on children and/or adult group [3,18,31,32]. These findings suggest that the immunologic reaction to H1N1 virus infection is associated with age and sex.

In this study, we found that clinical parameters such as admission rate, the frequency of underlying diseases, the rate of pneumonia and the values of laboratory parameter, such as CRP and ESR were associated with the increasing age. Especially, in the ≥ 65 y group (mean age 74 y), which showed significant difference compared to the other age groups all parameters. The ≥ 65 y group made only 0.9% of the total H1N1 virus infected patients (42/4,463). However, 5.6% of the admitted patients (21/373) and 9.5% of the pneumonia patients (11/116) were ≥ 65 y. It has been well documented that influenza can exacerbate underlying chronic disease, including asthma, chronic obstructive pulmonary disease (COPD), chronic hepatic or renal insufficiency, diabetes, or other cardiovascular conditions [6-9]. Given that a majority of H1N1 infected patients recovered without developing pneumonia, it is possible that the previously healthy patients that did develop progressive pneumonia may have had a hyperactive immunologic reaction to the viral insults [10-12]. However, because the ultimate recovery from viral infection should be controlled by immune system of the host, including T cells, we hypothesised that the patients with underlying diseases that require immune cells to control the diseases, may have an immunological defect as a result of immunological consumption. Patients with underlying diseases, especially older patients who have natural immune weakness, may experience a delayed recovery from viral insult or die from the deterioration of underlying diseases. Thus, it is possible that in these patient groups, the cause of mortality may be an individual immunological defect rather than viral infection. This phenomenon has been observed in other systemic infection including severe acute respiratory syndrome (SARS) by corona viruses and was recently observed in hemolytic uremic syndrome, which is caused by toxin-producing *E. coli *[33,34]. Furthermore, the proportion of these older patients (> 60 y) with underlying diseases accounted for > 50% of the fatal cases in Korea, despite the small number of total infections in this group (1.2% of the total infected patients) and in this study [9,35].

We previously reported that the children with pneumonia had higher leukocytes counts with lower lymphocyte differentials than the group without pneumonia, and that these parameters were associated with the severity of pneumonia [16]. In this study, we also found that the adult pneumonia patients had higher leukocytes counts with lower lymphocyte differentials, and higher CRP and ESR values compared to the group without pneumonia.

Because there have been no controlled-clinical trials that use corticosteroids to treat influenza patients since the last pandemic (1968 Hong Kong flu), the efficacy of corticosteroids on influenza infection is not known [36]. We previously reported that treating children with severe pneumonia with early corticosteroids effectively prevented disease progression [10,16,37]. In the present adult series, two previously healthy female patients (30 y and 42 y) had progress pneumonia and developed ARDS. Early infusion of corticosteroid (methylprednisolone) resulted in rapid recovery without mechanical ventilation therapy. The results of corticosteroid treatment on severely affected adult ARDS patients are also controversial [38,39]. It is possible that the numbers of patients with underlying diseases and different modalities for respiratory care among the study groups may be the major confounding factors with regards to mortality after treatment with immune modulators (corticosteroids) for patients with severe ARDS patients [38,39]. In addition, in this pandemic a part of pneumonia patients who were previously healthy presented with acute severe respiratory distress that was similar to an acute asthmatic attack [10]. Many of these patients may have been treated with early corticosteroids because it was assumed that they were having an asthmatic episode. Therefore, if these patients improved after corticosteroid treatment, they would not be included in the corticosteroid treated ARDS groups. The effect of corticosteroids on viral replication is controversial [40,41]. Additionally, it is unknown whether the virus particle itself or other mediators induce inflammation in lung lesions [10]. The corticosteroids may reduce the systemic immune reaction caused by hyperactive immune cells (T cells) with hypercytokinemia [10,38].

The initial lung injury that is caused by primary viral infection leads to secondary bacterial infection and further aggravation of ARDS [4]. Previous studies reported that 10-20% of the patients with severe pneumonia were co-infected with bacteria and that greater than 50% of the fatalities in pandemics were caused by co-infection with bacteria including *S. pneumoniae, S. aureus *and *S. pyrogens *[4,42]. These findings suggest that for previously healthy patients, early control of the initial acute lung injury may be essential to prevent ARDS and further lung injuries [10].

There are some limitations to this study. This retrospective observational study has a limited number of adult patients. Although our epidemiological data appeared to be similar to the nationwide data from the Korea government [9], the clinical data may not be a representative on 2009 H1N1 flu in Korea. There might be some differences on the policies of patient care, including admission and discharge, among clinicians in both departments. As for pneumonia patients, we did not perform extensive microbiological testing for other pathogens, we cannot rule out the possibility of co-infection with other respiratory pathogens.

## Conclusions

In 2009 H1N1 pandemic in Daejeon, Korea, the majority of infected patients (94%) were under 40 y, and males and females were equally affected. Pneumonia was observed in 2.6% of infected patents (116/4,463), and pneumonia was more predominant in male than female children, but more predominant in adult females than in adult males. The epidemiologic characteristics of this pandemic suggest that unknown herd immunity may have acted on the populations. The greater than 65 y group had the highest rate of underlying diseases and higher admission rate, pneumonia rate, total duration of fever, and CRP and ESR values than other age groups. These results indicated that these patients may have an immunologic defect that is associated with immunologic consumption by underlying diseases. Our results suggest that immunologic reaction of the host to viral insults is associated with age, sex and underlying diseases.

## Abbreviations

ARDS: acute respiratory distress syndrome; COPD: chronic obstructive pulmonary disease; CRP: C-reactive protein; ESR: erythrocyte sedimentation rate; RT-PCR: reverse transcriptase -polymerase chain reaction.

## Completing interests

The authors declare that they have no competing interest.

## Authors' contributions

KYL had primary responsibility for concept, design of the study and writing the manuscript; JWR participated in the preliminary data collection, data analysis and writing of the manuscript; EJG, YSY, MSK and SHP participated in patient care, data collection and data analysis; JHK contributed to editing of the manuscript and supervised the design and execution of the study; JCK participated in reading of chest radiograph of the patients. All authors read and approved the final manuscript.
